# Noise Levels and Acoustic Quality of Preschool Learning Spaces in Taiwan

**DOI:** 10.3390/ijerph23030406

**Published:** 2026-03-23

**Authors:** Wyatt H. Page, Stuart J. McLaren

**Affiliations:** School of Health Sciences, Massey University, Wellington 6140, New Zealand; stuart1mclaren@gmail.com

**Keywords:** noise levels, acoustic quality, preschool, early childhood centers, noise intrusion, noise management

## Abstract

**Highlights:**

**Public health relevance—How does this work relate to a public health issue?**

- Excessive noise exposure in preschool environments contributes to cognitive impairment, stress, and potential hearing damage in young children, a vulnerable population without coping strategies.
- Teachers also experience excessive occupational noise doses, linking classroom acoustics directly to workplace health and safety.

**Public health significance—Why is this work of significance to public health?**

- The study shows that adult-based noise standards are inadequate for protecting children, highlighting a regulatory gap.
- Findings provide evidence that poor acoustic quality in preschools is not just an educational issue but a broader public health concern affecting developmental outcomes and teacher well-being.

**Public health implications—What are the key implications or messages for practitioners, policy makers and/or researchers in public health?**

- Policymakers and practitioners should establish child-centered acoustic standards and implement architectural and behavioral noise-reduction strategies.
- Researchers should expand cross-cultural studies and develop evidence-based interventions to safeguard preschool environments worldwide.

**Abstract:**

This study examines noise levels and acoustic quality in preschool learning spaces in Taiwan, using a validated protocol from prior research in New Zealand. Seventeen classrooms across six centers, primarily in the major city of Kaohsiung, were assessed using acoustic measurements, personal dosimetry for teachers and children, and a structured teacher questionnaire. Most classrooms exceeded recommended reverberation time thresholds, and none met optimum acoustic standards. Fixed sound level meters substantially underestimated noise exposure during active periods compared to personal dosimetry. Dosimetry revealed that 24% of children and 27% of teachers experienced daily noise dose above 100% of the occupational criteria used in New Zealand and Europe. Notably, a center with a distinct educational philosophy demonstrated markedly lower individual exposure despite high ambient levels. These findings highlight systemic gaps in acoustic regulation for early childhood education in Taiwan and raise broader concerns about the default of using adult-based noise criteria for young children. Recommendations include Taiwan adopting or developing suitable acoustic standards and guidance values for noise levels in classrooms. The results have implications beyond Taiwan, offering evidence relevant to global efforts to improve preschool learning environments.

## 1. Introduction

Ambient noise has recently been described as the “new second hand smoke” [1] because of the wide range of adverse public health effects it causes. There is a wide range of definitions of noise, often dependent on the context. A common general definition is that “noise is unwanted sound”. In workplace or occupational settings, this often becomes “noise is all sound in the workplace, whether wanted or unwanted”, while for recreational settings, the definition emphasizes the voluntary nature of noise exposure. More recent definitions of noise, particularly from the European Union (EU) [2], emphasize its harmful nature. While no universal definition exists, noise in educational settings is typically understood as any unwanted or disruptive sound that interferes with teaching and learning. Excessive educational noise has significant negative impacts on both students and teachers. The 2011 WHO (World Health Organization) report on the burden of disease from environmental noise [3] identified cognitive impairment in children as a significant burden that was previously only suspected.

Noise in preschools or early education facilities is known to adversely affect children’s behavior [4]. Preschool children have not developed coping strategies to manage the effects of noise. This is exacerbated by the fact that noise levels in preschools are high in comparison to other school environments and less variable [5]. Society’s youngest children are exposed to some of the highest noise levels outside occupational settings.

A year-long study on noise and acoustics in early education facilities was conducted predominantly in the major city of Kaohsiung, Taiwan. The primary aim of the study was to assess the acoustic quality and noise exposure of teachers and children in preschool classrooms in Taiwan. The secondary aim of the study was to understand from teachers how noise affected them and the children they teach, what policies and procedures for noise were in place, and what strategies they used for managing classroom noise.

This study mirrored a large study in New Zealand by McLaren [6], undertaken over three years from 2003 to 2006 and used the same validated protocol. The results of the New Zealand study were influential in setting the noise criteria in the Licensing Criteria for Early Childhood Education & Care Services 2008 and Early Childhood Education Curriculum Framework [7], which are still in force today. The noise criteria are subjective, with an overall aim “to ensure that noise levels do not unduly interfere with normal speech and/or communication, or cause any child attending distress or harm.” The subjective nature of the criteria used to be a strength when most providers were government-operated. But today, like in Taiwan, the majority are private operators, with government providing subsidies per child, and the lack of objective criteria has proved to be problematic in achieving the original aim of the noise criteria.

The study “Noise and acoustics in early childhood education in Taiwan and effects on the children and their teachers” [8] was funded by a Taiwan Fellowship (Ministry for Foreign Affairs). The Research Center for Environmental Medicine (Kaohsiung Medical University (KMU)) hosted the second author for the duration of the study and provided logistical support and local facilities.

## 2. Materials and Methods

### 2.1. Approval

As the study involved young children and their teachers as participants, research ethics approval was required from the Institutional Review Board (IRB), the certified Research and Ethics Committee of the Kaohsiung Medical University Chung-Ho Memorial Hospital in Taiwan.

The IRB was supportive of the project but raised specific concerns about the equipment (a personal sound exposure meter or dosimeter), which was to be pinned to the clothing of children in education facilities, where parental consent had been obtained, and oral assent had been given by the children. The material safety data sheets from the manufacturer were submitted to the IRB regarding the safety of the devices. Approval was granted in April 2019 and covered the study protocol, informed consent forms and a questionnaire, both in Mandarin and English. Oral assent from the children was obtained by the teacher showing them the dosimeters, explaining what they did, and demonstrating it being attached. The novelty of wearing the dosimeter and not wanting to miss out meant that for some classroom sessions, a few “dummy” units (they look the same as the real thing but have no electronics inside) were used to ensure every child with parental consent who wanted to participate was able to.

### 2.2. Recruitment

Recruitment in Taiwan proved considerably more challenging than in the earlier New Zealand study, where nearly universal participation was achieved. Despite outreach via formal letters and in-person visits, fewer than 10% of contacted facilities agreed to participate. Several factors likely contributed to this low uptake.

Language barriers were a notable obstacle. The lead researcher, not fluent in Mandarin, was accompanied by an interpreter throughout the study. While this arrangement generally functioned well, it posed limitations in building trust with facility staff and administrators. In one instance, when the preschool director discussed the study with the teaching staff, they initially received strong negative feedback that the study was setting out to criticize them and find fault. Staff apprehension was evident during the first measurement session, though rapport and trust improved following positive feedback and successful sessions. Two centers that initially consented later withdrew due to management changes. Additionally, some stakeholders appeared to perceive the project as a medical study, which may have heightened hesitancy. This aligns with findings from a Taiwanese study [9] on research participation, which emphasized the importance of securing approval from both institutional authorities and individual participants, such as teachers.

The research aimed to investigate about 40 classrooms, as most preschools have several classrooms or learning spaces. However, due to recruitment challenges and time constraints, only seventeen (17) learning spaces from six early childhood centers were involved. Most were from the major city of Kaohsiung. These primarily included privately operated centers, as well as one public center embedded in an elementary school, and a special character center (such as those subscribing to the educational philosophies of Maria Montessori [10] or Rudolf Steiner [11]).

Although no demographic or socioeconomic information was collected about the parents of the children in the participating centers, observationally based on the level of equipment in the centers and the quality of the buildings they occupied, it is likely they covered a wide variety of socioeconomic status. It should be noted that at the time of this study, about 58% of children 2 to 5 years were enrolled in preschools in Taiwan, whereas of mid-2024, this had increased to around 82% due to changes in government policy [12]. In New Zealand, over the same period, it is difficult to calculate comparative values for the same age group. However, participation in preschool in New Zealand has been high and stable for a long time, with participation of children six months prior to entering school very high at 97% [13].

### 2.3. Legal Requirements

The legal requirements, standards and guidelines for preschool education and workplaces in Taiwan and New Zealand enacted at the time of this study were considered. In both jurisdictions, the legal requirements for preschools are contained in principal legislation, which is underpinned by standards and criteria.

In Taiwan, the principal legislation is the Early Childhood Education and Care Act [14]. Underpinning this are the standards of basic facilities and equipment for kindergartens and their classes [15]. These have been promulgated pursuant to Article 8, Item 6 of the above Act, and were in force at the time of the study.

In New Zealand, at the time of this study, the Education Act 1989 was the principal Act under which the Education (Early Childhood Services) Regulations 2008 [16] were made pursuant. The Licensing Criteria for Early Childhood Education and Care Services 2008 and Early Childhood Education Curriculum Framework [7] were promulgated under the 2008 regulations.

In the Taiwan regulations (Article 16), there is no limit on the total number of children that can be enrolled in any one facility. Preschools for children 2–3 years old are limited to 15 children per class and cannot be mixed with children of other ages. For children over 3 years old, class numbers are limited to 30 children per class. In New Zealand, a maximum of 25 children under 2 years of age may be enrolled in any one center or facility. For those over 2 years of age a maximum of 150 children may be enrolled without approval of the “Secretary of Education”. No more than 50 children (of mixed age) can attend at the same time (without secretarial approval). The “Secretary of Education” may approve a maximum of 150 children of mixed age, with the provision that no more than 75 children under 2 years old may be enrolled. Services must ensure all children will be adequately catered for and the different needs of children attending will be met.

#### 2.3.1. Space Requirements

In the Taiwan regulations, Article 10 specifies the indoor activity room space requirements of a minimum of 2.5 square meters per child, the same as the New Zealand criteria. Both jurisdictions also have a requirement for outdoor space. In Taiwan, it is a minimum of 3.0 square meters per child, whereas it is 5.0 square meters per child in New Zealand. However, the “outdoor” activity space in Taiwan is typically the ground floor (or second or third floor) of the early childhood center building, whereas in New Zealand, it will most commonly be an outdoor space adjacent to the building containing the indoor space.

#### 2.3.2. Noise Criteria

New Zealand has an acoustics standard for the performance of building interiors [17], such as classrooms. It covers the internal ambient or background noise levels and reverberation time. These are assessed when the classrooms are unoccupied and not in operation. Taiwan has a series of acoustics standards, but their focus is on sound insulation, reducing the transmission of sound between spaces. It does not have a standard for the acoustic performance of building interiors.

In Taiwan and New Zealand, like elsewhere in the world, there are no standards for noise exposure of children in classrooms and learning spaces while they are in session and carrying out their normal activities. Often, as a starting point, researchers have used workplace noise criteria for children in educational settings, as this applies to the classroom teachers. Such criteria are designed for adults, typically based on an 8-h workday, and assume the remaining 16 h of the day have minimal noise exposure. Universally, workplace noise regulations use a two-part criterion. The first part covers the continuous part of the noise exposure, with a limit set using the A-weighted time-average level (or time weighted average) over 8 h (L_Aeq(8 h)_, see the Abbreviations and definitions section). The second part of the criterion is designed to reduce the additional risk of hearing damage from impulsive or explosive sounds. This uses the maximum instantaneous sound pressure level, typically 140 dB L_Cpeak_ (C-weighted) in most jurisdictions.

Workplace noise criteria are set to minimize long-term hearing damage but not eliminate it. For example, the New Zealand standard for occupational auditory assessment [18] predicts a 10 dB hearing loss for 95% of a noise-exposed population if they are exposed for 40 years to the acceptable maximum time average level in their workday. However, it is recognized by hearing specialists that children are likely to suffer hearing damage if exposed to noise at workplace criteria levels, even for relatively short durations. A review of speech interference in classrooms [19] stated that acute cochlear damage can occur in children’s hearing, while the same level will have no such effect in adults.

The workplace continuous noise criterion can be expressed as a percentage dose. A workday of 8 h duration at this noise level corresponds to 100% dose. This is calculated by taking the time-average level off the dB scale to get its Pa^2^ value. This is then multiplied by the time to get the noise exposure. Using the workplace criterion of 85 dB L_Aeq(8 h)_, this becomes 1 Pa^2^ × 8 h = 1 Pa^2^h. This in turn can be expressed as a percentage, with = 1 Pa^2^h equal to 100% dose. However, across different jurisdictions, there are different ways to calculate noise dose. New Zealand uses the same international workplace noise criteria as the European Union. The Taiwan workplace noise criteria are the same as in the United States, with higher criterion levels and different exchange rates. The effect of this is that 100% dose using the Taiwan criteria corresponds to 320% dose using the New Zealand criteria. To avoid confusion when comparing the results to those of McLaren [6], the New Zealand criteria will be used when calculating noise dose, and they will be grouped into the following three dose bands: <50%, 50–100%, and >100%.

### 2.4. Assessment

There were three elements to the assessment of each participating preschool center.

1. Acoustic assessment: This consisted of a visual inspection of each learning space, noting the construction, the internal surface (floor, walls, ceiling, and windows) finishes, the presence of any acoustic materials, and any noise-generating equipment such as an HVAC (Heating, Ventilation, and Air Conditioning) system. Room dimensions were taken so the volume could be estimated for acoustic analysis. With the rooms unoccupied, the reverberation times (an important metric for acoustic quality) were measured in octave frequency bands in the middle of the rooms using a “01 dB Solo” sound level meter set to “T60” mode. This measurement feature implements the process described in ISO 3382:1997 [20]. Measurements were repeated three times and averaged to get a robust estimate.
2. Noise measurement: Three types of noise measurement were carried out, and they were repeated twice for each classroom.
  (i) General classroom noise—In each learning space, a 01 dB Solo sound level meter was mounted on a tripod at ear height (1.5 m), away from walls and other reflective surfaces, but positioned so as not to interfere with normal movement in the learning space. It provided measurement of the general classroom noise levels while in operation, but not directly at the main noise sources (teachers and children) locations, which often moved around the space.
  (ii) Teacher dosimetry—A personal sound exposure meter (Cirrus Research original doseBadge [21]) was fitted to each teacher in accordance with the manufacturer’s instructions. They wore them throughout the day, including the sleep period, where they remained with the children.
  (iii) Child dosimetry—A personal sound exposure meter was fitted to selected children (those with parental informed consent). They were worn during children’s daily activities, except during the sleep period, when they were placed on a table/bench close to the children. They were refitted to the child once they were awake.
3. Questionnaire: At the end of the final measurement session, the teaching staff were invited to complete a simple, confidential questionnaire (available in both Mandarin and English). The questions canvassed aspects of their teaching space, teaching practice in managing noise generated from activities, teaching practice and intrusion from noise sources outside the center. Respondents could also choose the questions they answered. The Questionnaire is available as Supplementary Material to this manuscript. The translation from English to Mandarin was carried out by staff in the Research Center for Environmental Medicine at Kaohsiung Medical University (KMU). The initial translation was piloted with a school teacher, and minor changes were made to improve clarity before final approval was granted by the Institutional Review Board at KMU.

Note: All sound measurement equipment held a current laboratory certification and was calibrated before starting measurements and checked after each measurement was completed. No variation outside the acceptable range occurred.

## 3. Results

### 3.1. Acoustic Assessment

Table 1 summarizes the results of the acoustic assessment. The centers are numbered 1–6 to preserve anonymity. Most centers had more than one classroom, and in all but one center, all classrooms were assessed.

The final column of the table rates the spaces using the reverberation time criteria for learning spaces in the Australian/New Zealand standard AS/NZS 2107:2016 [17]. These criteria are similar to those in other international standards. Only seven of the 17 classrooms were compliant with the “good” reverberation criteria, and none met the “optimum” criteria, a very similar proportion to the New Zealand study [6].

Most classrooms consisted of polished wooden floors, plastered walls and suspended ceiling tiles, with no acoustic treatment or soft furniture. One center in a relatively new, well-appointed building had hard reflective floor, wall and ceiling surfaces and no acoustic treatment. In the center, which was part of an elementary school, the three preschool spaces were located off an open corridor with no physical barrier separating them from the corridor. The other side of the rooms directly faced the play area, where the older children would play during their break time, disturbing the preschoolers who were taking their afternoon sleep.

### 3.2. General Classroom Noise Levels

A typical day for children in preschools in Taiwan consists of arrival, usually between 8.30 and 9:00 am. Throughout the day, the children are involved in a variety of learning activities, including reading and writing in Mandarin and simple English, arithmetic and play. In some centers, they will leave the classroom to engage in sports and play outside. A cooked lunch is served at all the centers, after which the children sleep for 1–2 h in the early afternoon. Following their rest, they engage in further learning activities until departure, usually between 3:00 p.m. and 4:30 p.m.

**Figure 1:**
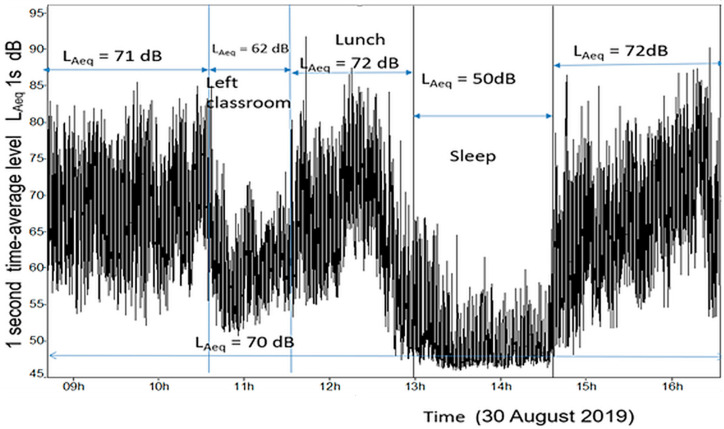
Time history of the general noise levels in a classroom in Center 1, across a typical preschool day, segmented by activity.

Figure 1 shows a time history of the time-average noise levels over a day for one of the classrooms in Center 1, measured using a fixed sound level meter. The levels are sampled at one-second intervals. This time history is representative of many of the classrooms in this study and is segmented by classroom activity.

The first period, from 8:45 to 10:00 a.m., is an active time, with a time-average level of 71 dB L_Aeq(T)_ (see the Abbreviations and definitions section). They then leave to exercise outdoors away from the center, and the sound levels drop to an average of 62 dB L_Aeq(T)_. They return at about 11:30 a.m. and have lunch from 12:00 to 12:45 p.m. During this period, the average level was 72 dB L_Aeq(T)_. The sleep period is the quietest part of the day, with an average level of 50 dB L_Aeq(T)_. The final period is normal active time, with an average level of 72 dB L_Aeq(T)_. In the last 10 min of this period, the levels drop markedly as the teachers prepare the children for pick-up by their parents.

Table 2 summarizes general classroom sound measurements across the centers. The first column is the time-average level range across the whole day (7, 7.5, or 8 h) for the classrooms in the center. The second column is the time-average level during the sleep period. The third column corresponds to the total active time of the day, when children play, often noisily, and show the highest noise levels. The noise levels varied between classrooms in the same center, across centers, and for repeat measurements. The lowest and highest full day time-average levels were 70 and 81 dB respectively and the mean over all classrooms was 74 dB. Centers 4 and 5 have the lowest levels, most likely due to their compliant, but not optimal reverberation time. The largest variation in the full day time-average level occurred for Centers 2 and 3, which had the highest reverberation times. As expected, the sleep time time-average levels were much lower (by about 20 dB) than during the active time, averaging about 52 dB L_Aeq_. The exception was Center 2, which was about 10 dB higher due to the intrusion of noise from nearby traffic outside the building. If good sleep was the focus of the sleep period, levels below 40 dB would be advantageous.

### 3.3. Dosimetry—Teachers and Children

All dosimetry was observed by a researcher to ensure there were no confounding issues, such as tampering with or purposely shouting into the personal sound exposure meters. Contemporaneous observational notes were made by the researcher for each measurement session, with a description and approximate timing for the classroom activities.

**Figure 2:**
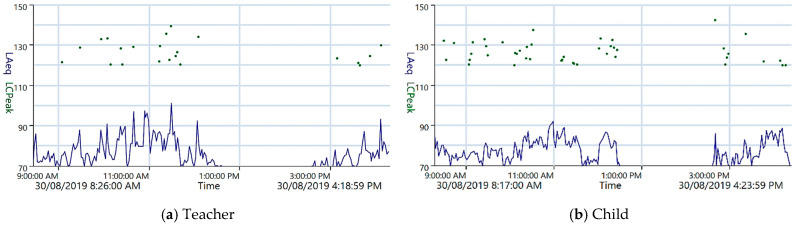
Time histories of noise levels from dosimeters for the same day in a classroom in Center 1. The blue trace represents the time-average level, while the dark green dots towards the top indicate the peak exceedances (≥120 dB).

Figure 2 shows example time histories of the noise levels for a teacher and a child on the same day in a classroom in Center 1. The personal sound exposure meters produce a time history of time-average levels at 1 min intervals, with up to 93 peak levels stored if they exceed 120 dB L_Cpeak_. As is common with personal sound exposure meters, levels below 70 dB L_Aeq_ are not recorded. The 70 dB cutoff is most obvious during quiet periods such as sleep time, starting at about 12:45 p.m. This cutoff introduces uncertainty in the percentage dose of a maximum of 3% over an 8 h day, as 70 dB L_Aeq(8 h)_ is equivalent to about 3% dose. However, in practice the uncertainty due to the cut-off is likely to be significantly less as the quiet periods are only about a quarter of a typical day.

The teacher in this session is instructing children, often in a loud voice that at times exceeded 90 dB L_Aeq(1 min)_, sometimes reaching 100 dB, whereas for the children, it rarely exceeded 90 dB. For peak noise levels, there were many exceedances of the 120 dB L_Cpeak_ recording minimum for both teachers and children, with one for a child exceeding the workplace criterion (140 dB). Based on the observational notes, this peak exceedance occurred when two metal chairs came into contact. The other recorded peak values typically correspond to physical activities, some involving toys or musical instruments such as drums.

Table 3 summarizes the noise dose of teachers and children across the centers. In all centers, at least one teacher participated (15 in total), and in all classrooms, there was at least one child participating (83 in total), with some centers having significant numbers involved across their classrooms. The second column in the table is the percentage noise dose range calculated from the full day time-average level ranges for the general classroom noise in Table 2. These represent the percentage dose the researcher was exposed to when observing the class sessions. The three percentage dose bands in Table 3 are the same as those used in the New Zealand study [6]. In this study, exposure of 50% or less was deemed “not to be of concern”. Levels 50–99% were of “significant concern”, and those exceeding 100% were of “serious concern”. As the dose bands are broad, no adjustment was made to account for the 70 dB cutoff of the dosimeters.

The general percentage noise dose is much lower than the values of the dosimeters. The main reason for this is the proximity of the dosimeter to the noise sources. In accordance with best practice, the dosimeters were attached to the participant’s clothing within 10–20 cm of the ear. This proximity increased exposure readings, particularly from the participant’s own voice and nearby activity. This effect is particularly noticeable for the teachers from Center 2, where all three had a dose exceeding 100%. These teachers wore portable hip-mounted PA systems that they often used to try to make themselves heard against the poor acoustics of their classrooms. In practice, this did not improve the intelligibility of their voice (as noted by the translator accompanying the researcher) and was so loud at times that it was uncomfortable due to their narrow frequency bandwidth and high output levels.

Just over half (53%) of the teachers were in the lowest percentage dose band (<50%). For children, only about a third (34%) fell into this band. A fifth (20%) of the teachers were in the middle band (50–100%), while 38% of the children were in this band. In the top band, those greater than 100% dose, the teachers from Center 2 that used the portable PA systems, dominated. A considerable portion (22%) of children were also in the top band, with 40% from Center 2 and the remainder spread across the centers, with none from Center 6.

The Center 6 results are notable, with teachers and children all in the less than 50% dose band, with the actual values for children being less than 25% dose. The classrooms in this center were not quieter than the other centers, as indicated by the general full-day time-average level in Table 2 of 77 dB, which is one of the highest. However, due to the special character of the teaching philosophy of this center, emphasizing self-control and self-direction, with minimal ordering from teachers, or shouting from children, the individual percentage dose measured by dosimetry, were markedly lower than the other centers. The closest match to this center in the New Zealand study in terms of philosophy and results are the “Playcentres” [22]. This type of preschool center is relatively unique internationally, having first started in New Zealand in 1941. The underlying philosophy is parents as teachers (parents are required to complete early childhood accredited learning modules), allowing them to support and watch their child’s confidence grow as they explore and foster their imagination through play and interaction with other children. This supportive play-focused environment is often noisy, but because it emphasizes self-control and self-direction regulation, it is rare for children to shout or raise their voice, except perhaps during indoor singing or when engaged in outdoor play.

### 3.4. Questionnaire

The anonymous teacher questionnaire was distributed in paper form and responded voluntarily. The response rate was very high (93%), with only one teacher choosing not to complete it, with 14 participating in total. The questionnaire was in seven parts, with each part consisting of a set of structured questions. Most questions required a textual response, and the responses were grouped by them. Part 1 focused on the teaching spaces and asked them to rank the following aspects on a five-point scale, where “5” is the most important and “1” the least:

- Lighting;
- Ventilation;
- Acoustics (listening environment);
- Equipment;
- Space.

Also, in this part, they indicated the quality of their experience of the acoustics and listening environment for each of the classrooms in their center. Part 2 of the questionnaire focused on the noise generated in their classrooms, the sources, when it was perceived to be excessive, and what portion of the working day they considered the noise level to be too high. Part 3 covered noise sources from outside their classroom, including from other classrooms and from outside their center. Part 4 focused on their perception of how classroom noise affected their children. Some of the centers in the study had children with special conditions, needs or disabilities. Part 5 of the questionnaire focused on whether teachers have experience working with children with disabilities that are known to be affected by noise. Part 6 looked at how noise affected their teaching. The last part (7) considered policies, procedures, and strategies for managing noise, and included several open-ended questions.

**Figure 3:**
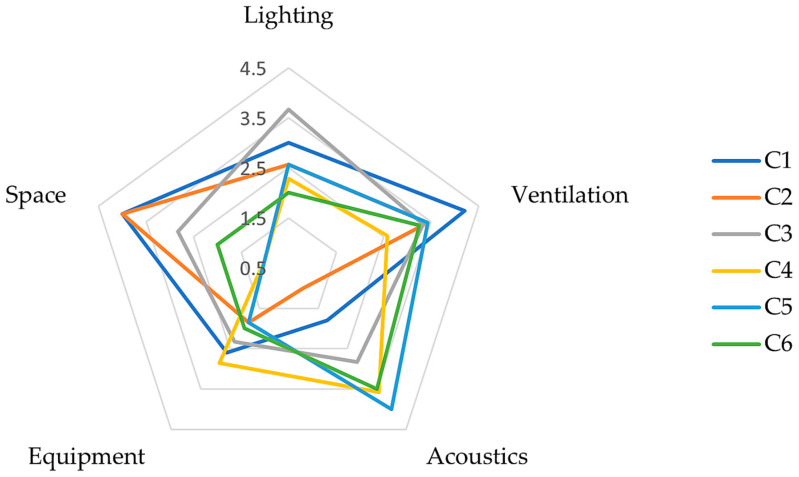
Ranking of importance of classroom physical aspects (5 = most important, 1 = least important).

Figure 3 presents the results of the ranking of the five aspects of the classrooms from Part 1 of the questionnaire. The individual teacher rankings were aggregated for each center and averaged to produce an overall rank for the center. As expected, there are differences between the aspect rankings across centers. Good lighting and ventilation were generally ranked with a 3 or higher, with ventilation being the top rank for Center 1. Half of the centers ranked acoustics as a major priority with a rank 3.5 or higher. Only one center ranked acoustics as its lowest priority. For most of the center’s equipment was not a high priority, they indicated that they were adequately resourced in this area. Two centers ranked floor activity space as their top priority.

The rating of the classroom listening environment used a four-point scale as follows: “Very Poor”, “Poor”, “Good”, and “Just right”. Ratings varied from poor to good, with all teachers saying their spaces have too much echo (reverberation), and with some describing listening as confusing, harsh and unclear.

In Part 2 responses on noise generated in their classrooms, all teachers indicated that the main source of classroom noise was the children and their activities. The start of the day, free play times, lunch time, and the end of the day when parents arrive to pick up their children were identified as the noisiest periods. All teachers said there was a portion of the day in which they rated the classroom noise as excessive. Typically, this was about 20% of the day, but for the centers with a low rating of their listening environment, this could be as high as 60% of the day.

In Part 3 responses on noise sources outside the classroom, many teachers indicated the intrusion of noise from outside the classroom. For half of the centers, this noise was from adjacent classrooms, while for others it was external to their building. Typically, this included road traffic noise, noise from emergency vehicles, and construction noise. The three centers with ground-floor classrooms specifically noted promotional vehicles passing by advertising their message with loudhailers, as a regular problem that was a nuisance.

Part 4 of the questionnaire focused on teachers’ perception of how classroom noise affected their children and consisted of five structured questions. A very common response was that when the noise rises, the children become excited (“it hypes them up”), and the “noise rises more and gets to a point where they are unable to concentrate”. This phenomenon of rising noise is called the Lombard or café effect [23] and is particularly problematic in classrooms with poor acoustics, where the sound is not effectively absorbed. Teachers also commented that at the end of the day, when children were tired, noise made them irritable.

Part 5 of the questionnaire consisted of six structured questions. In the first question, teachers indicated from a list of disabilities and special needs the ones they had experience with and in the following questions characterized how noise affected these children. Over half of the teachers said they had worked with children with ADHD (Attention-Deficit/Hyperactivity Disorder) and some with ASD (autism spectrum disorder). All respondents felt that ADHD and ASD children were more adversely affected by noise than other children. Common themes in the answers were that it increased the child’s anxiety and caused them to withdraw, that they were not easily settled in the presence of noise and would become agitated, and in some cases, scream. Overall, they were more easily distracted by all sources of noise.

When responding to the structured questions in Part 6 of the questionnaire on how noise affects their teaching, common themes included that it was irritating, a nuisance, and it created a negative mood, sometimes resulting in them losing patience with the children. Several commented that voice strain was an issue and that classroom noise was possibly affecting their hearing.

The final part of the questionnaire consisted of seven structured questions requiring a textual response. Many of the questions were open-ended and allowed teachers to comment on policies, procedures, strategies for managing noise, and any area where they felt further work was required or they needed help. None of the respondents indicated that their center had any formal policies for managing noise. One teacher suggested that a noise education program be developed for children and their parents. For children, this could include instruction and guidance on how loudly they should speak and manage and being aware of the noise they make. Another teacher suggested that there should be training and development of skills and methods for teaching staff in the minimization of classroom noise. A range of strategies were used by teachers to manage classroom noise. These included quiet times, banning activities which generate excessive noise, and using a calm voice to ask children to sit with their hands on their knees until they settle down. Many commented that raising their voices was not an effective strategy to manage noise. A common request was advice on improving the acoustics of their classrooms and if there were any low-cost retrofit solutions. Similar requests were made in the response to these questions in the New Zealand study. But at the time, there was little suitable and accessible guidance available. With the development of the “Designing Quality Learning Spaces” series of documents for classrooms, which includes acoustics [24], we were able to provide them with a copy and supply links to acoustics product in New Zealand that have proved to be cost-effective to retrofit and reduce the reverberation time. We then advised them to look for similar products in their own market.

## 4. Discussion

The findings of this study closely align with those of a comparable investigation conducted over a decade earlier in New Zealand, which employed the same protocol. Measured reverberation times, noise levels and percentage noise dose, as well as questionnaire responses, were similar.

The major city population densities in Taiwan are at least twenty times that of New Zealand; these cities are far noisier [25]. Thus, there is an expectation that this higher environmental noise would have an impact on the results of noise in preschool classrooms. There are several reasons why this is not the case. Firstly, because of the high population densities, Taiwan has a well-established noise control act with stringent environmental noise protection ordinances [26]. This is supported by modern building codes and acoustic regulations for residential and hospitality buildings. The acoustic regulations focus on sound insulation, minimizing the intrusion of noise from outside the building or from other occupants within a shared building. However, like New Zealand, Taiwan does not have regulations for the indoor acoustics of buildings, that is, how well noise made within a space is absorbed. The Ministry of Education in New Zealand developed the “Designing Quality Learning Spaces” series of documents, which includes acoustics [24]. New build school projects from 2017 must comply with the mandatory requirements outlined in these documents. However, they do not apply to private preschools as they are not government-owned. Also, because of hygiene reasons, most surfaces in preschools are hard and easy to clean. Such surfaces reflect sound well, increasing the reverberation time and general classroom noise levels.

The general classroom noise levels measured by fixed sound level meters showed that preschool classrooms are noisy. The time-average levels over the day were typically 72 dB L_Aeq_, with short-term levels regularly exceeding 85 dB. These results are similar to those reported in other studies of preschool classroom noise [27]. All these studies positioned the sound level meter on a tripod at a height of 1.5 m or similar above the floor. The likely reason for this is that this is the recommended height for occupational noise assessment of a standing adult. The recommended noise measurement height for seated adults might be considered more appropriate in this setting. However, this is still significantly higher than the ear height of a standing four-year-old at about 0.85 m. The implications in terms of the noise levels measured at a height of 1.5 m are twofold. Firstly, the levels are likely to be lower because they are taken above the height of the plane, where most of the noise is created by the children and their activities. The levels will also be lower because they are further away from the nearest reflective surface, the floor, and closer to the main absorbing surface in most of these classrooms, an acoustic tile ceiling. Estimates of noise dose based on these general classroom noise measurements will underestimate the exposure.

Very few studies have performed noise dosimetry, that is, measuring directly the noise exposure of individual children and teachers in their classrooms. This is important as most of the noise in preschools is created by individuals (children and teachers) and their activities. The dosimetry results show that using the general classroom noise levels will underestimate the individual noise exposure. For teachers, the underestimation was two to five times, corresponding to a difference of 3 to 7 dB L_Aeq(T)_, while for children, it was even higher (three to ten times), corresponding to a difference of 5 to 10 dB. Alarmingly, just like in the New Zealand study, almost two-thirds of the children had an exposure exceeding 50% dose, with almost a quarter exceeding 100%. Given that the exposure criteria were designed for adults in the workplace to limit hearing damage, exceeding such levels in a preschool environment with very young children is highly problematic.

The teacher questionnaire highlighted that for many centers, teachers ranked acoustics (“decreasing the echo”) as a high priority, indicating that they understood the importance of good indoor acoustics in their classrooms. However, teachers from one center did not understand that increasing the level of their voice by using hip-mounted portable PA systems in classrooms with poor acoustics only made the situation worse. Across all centers, teachers said that for a significant portion of the day (20–60%), classroom noise was excessive. This subjective assessment can be applied to the noise level measurements to estimate the level at which teachers considered the noise excessive. For classrooms where teachers agreed that 20% of the time, noise was excessive, this corresponds to a level of about 75 and 84 dB L_A20,_ respectively, based on general classroom noise measurements and teacher dosimetry. For classrooms where teachers agreed that 60% of the time the noise was excessive, the estimated levels (L_A60_) were very similar to the 20% of the time case, but the reverberation time for these classrooms was noticeably longer (T_mf_ = 0.8 compared to 0.6 s). This finding emphasizes the interplay between measured noise levels and reverberation time when rating the noise as excessive. Generally, the level at which noise was rated as excessive decreased with increasing reverberation time because of the loss of speech intelligibility. The description from teachers about how noise adversely affected children was consistent with other studies [4] where reported noise levels were comparable to those measured in this study.

A major source of irritation and noise nuisance that was not noted in the New Zealand study and other international studies, but was regularly mentioned in this study, was advertising noise from mobile vehicles. For preschool centers that were located on busy streets, it was common for classroom sessions to be interrupted with audio advertising of commercial or political messages (it was an election year during the study) from vehicles that would stop outside, often for extended periods. Such advertising is an approved but restricted commercial activity in large cities in Taiwan by local bylaws. Similar bylaws exist in most countries, including New Zealand. The use of “sound trucks” is common in high population density cities in many countries, including Japan, India, and Brazil. In principle, a complaint can be made to the local authorities about these vehicles, but because of their transient and unpredictable appearance, it is not usually practical to make a complaint.

An important gap identified by teachers was in the education of children, their parents, and teachers about the adverse effects of noise. Even though parents and teachers generally understand that preschool children are a vulnerable group, they tend to use an adult lens when considering what is acceptable noise. This is compounded by the fact that none of the centers had noise management plans. Specific guidance on managing activity noise in preschool settings has been developed with a New Zealand perspective [28], but is likely to be similar for international settings. As part of this, a distinction is made between activities that require quiet and concentration and those that generate noise. Rather than outright banning noisy activities (as some teachers suggested in this study), many can be modified to reduce the noise level while still allowing enjoyment and full participation. A recent scoping review on the effect of classroom acoustics and noise on preschool children’s listening, learning, and wellbeing [29] said research is still needed to determine the optimum classroom acoustic parameters and noise levels, while allowing noise that is necessary. This review also highlighted the importance of teacher awareness of when noise is too high and is likely to impact children’s listening, learning, and wellbeing, so it can be managed. Noise control in preschool environments requires an interdisciplinary approach due to the complex functions and interactions that occur, and preschool education’s dual role of both education and childcare.

The most significant limitation of this study is the sample size and representativeness of the preschool centers, given the low recruitment rate. This is likely to limit the generalizability of the findings. The lead researcher visited many preschool centers in both Kaohsiung and Taipei after the initial extremely low recruitment rate from using email and a formal letter. He spoke (with the aid of a translator) to center managers and teachers about the importance of acoustics and managing noise in their classrooms and that he had carried out a similar study in New Zealand. This in-person approach lifted the recruitment rate and provided an opportunity to see a wide range of centers and the facilities. In general, those that declined to participate could subjectively be matched to one of the centers that did participate, in terms of size, number of classrooms, and type of location. So, although the number of centers in the study was low, they are likely to be representative of the variety of preschool centers in Taiwan. Only seventeen classrooms were assessed in this study, representing all the classrooms in the centers that took place. In almost all centers, at least two all-day noise measurements sessions were performed to help ensure the data were representative.

## 5. Conclusions

This study provides one of the first comparative assessments of noise levels and acoustic quality in preschool learning environments in Taiwan, with reference to established benchmarks and prior research in New Zealand. The findings reveal that:

- Acoustic conditions in Taiwanese preschools are generally suboptimal, with most learning spaces exceeding recommended reverberation times and lacking adequate acoustic treatment.
- Noise levels from fixed sound level meter in classrooms underestimate the actual exposure when compared to dosimetry.
- Noise exposure for both teachers and children frequently exceeded occupational health criteria, with about a quarter of both groups experiencing daily noise doses above 100%, levels that are associated with long-term auditory risk.
- Center-specific factors, including teaching philosophy and classroom design, significantly influence individual noise exposure, as evidenced by the notably lower dosimetry results in the special character center, despite high ambient levels.

These results underscore the need for national standards in Taiwan that address internal acoustic performance in early childhood education settings, beyond current structural and insulation-focused regulations. Adoption of a standard like AS/NZS 2107 [17] and the development of classroom acoustics guidelines, perhaps modeled on the New Zealand one [24], would be a good starting point. The results also highlight the limitations of applying adult occupational noise criteria to children, whose vulnerability to auditory and cognitive impacts is far greater. In the McLaren [6] study, below 50% noise dose was deemed “not to be of concern”. However, this still corresponds to a time-average level of about 82 dB L_Aeq_ over a typical session time (8 h). Considering auditory effects alone, the time-average level should be limited to 75 dB L_Aeq(8 h)_. This is equivalent to 10% dose (occupational criteria), and is also the same as the 2018 WHO conditional guidance value for leisure noise [30]. When cognitive, learning, and wellbeing effects are considered, a level below 70 dB L_Aeq(8 h)_ would be more appropriate with a target of 65 dB not being unrealistic. In contrast, normal adult conversation speech levels are typically 60 dB L_Aeq_ at 1 m.

Future research should explore the longitudinal effects of chronic noise exposure on preschool children’s development and wellbeing, and evaluate the efficacy of interventions such as acoustic retrofitting, teacher training, and pedagogical adaptations. Dosimetry is not likely to be practical for long-term studies, and so general classroom noise measured with a fixed sound level meter height should be made at 0.8 m above the floor, close to the ear height of a standing four-year-old. Also, based on the findings for Center 6, pedagogical adaptations potentially could produce valuable gains in reducing individual (teachers and children) noise exposure. Studies, like this one, can inform evidence-based policy harmonization and support the creation of healthier, more equitable learning environments for young children.

## Figures and Tables

**Table 1:** Description of individual learning spaces and acoustic quality.

Center	Classroom		Mid-Frequency Reverberation Time, T_mf_ * (s)	Room Character Notes	Reverberation Criteria (s) Good: 0.4–0.6 Optimum: 0.3–0.4
	Count	Volume (m^3^)			
1	3	125	0.7 (all rooms)	Floor and walls are hard reflective surfaces, with suspended ceiling tiles.	Non-compliant
2	3	100–150	0.8 (all rooms)	All hard reflective surfaces.	Non-compliant
3	3	Not determined	0.8 (two end rooms) 0.9 (middle room)	Three rooms in quasi open-plan layout. All hard reflective surfaces.	Non-compliant
4	3	225	0.6 (all rooms)	Floor and walls are hard reflective surfaces, with suspended ceiling tiles.	Compliant, but not optimum
5	3	130	0.6 (all rooms)		Compliant, but not optimum
6	1	41	0.6	Polished wooden floors, exterior walls, all hard plastered concrete with wood paneling to 1 m and windows. Internal walls with glass viewing windows, suspended ceiling tiles.	Compliant, but not optimum
6	1	100	0.7		Non-compliant

* T_mf_ is defined in Section 5.4 of AS/NZS 2107:2016 [17] as the average of the reverberation time values in the 500 Hz and 1000 Hz octave bands.

**Table 2:** General classroom noise levels in each center.

Center	Full-Day, Time-Average Level, L_Aeq(T)_ (dB)		Sleep Time, Time-Average Level, L_Aeq(T)_ (dB)	Active Time, Time-Average Level, L_Aeq(T)_ (dB)
	Range [Time]	Mean [SD]		
1	70–73 [7.5 h]	72 [1.2]	50 [T ≈ 1.5 h]	72–79 [T ≈ 6 h]
2	73–81 [7.5 h]	76 [2.7]	60–64 [T ≈ 1.5 h]	79–82 [T ≈ 6 h]
3	71–77 [7 h]	75 [2.4]	51–53 [T ≈ 1.5 h]	71–77 [T ≈ 5.5 h]
4	68–73 [7.5 h]	71 [1.1]	53–54 [T ≈ 1.5 h]	71–74 [T ≈ 6 h]
5	71 [8 h]	71 [<0.5]	50–54 [T ≈ 1.5 h]	65–73 [T ≈ 6.5 h]
6	77 [7.5 h]	77 [<0.5]	Not measured due to characteristics of the school	
**Summary**	70–81 [7–8 h]	74 [1.6]	50–64 [T ≈ 1.5 h]	71–82 [T ≈ 6 h]

**Table 3:** Noise dose exposure.

Center	General (%dose)	Teacher Count	Teachers in %dose Range			Child Count	Children in %dose Range		
			<50%	50–100%	>100%		<50%	50–100%	>100%
1	3–6	2	2			14	6	4	4
2	6–38	3	0		3	22	4	10	8
3	4–14	4	1	3	0	21	10	8	3
4	2–6	2	1		1	18	5	9	4
5	4	1	1		0	5	1	3	1
6	15	3	3		0	3	3	0	0
**Totals**		15	8	3	4	83	29	34	20

## Data Availability

The data presented in this study are available on request from the corresponding author due to ethical restrictions on their use.

## Supplementary Materials

The following supporting information can be downloaded at https://www.mdpi.com/article/10.3390/ijerph23030406/s1. File S1: Questionnaire.

## Abbreviations and Definitions

The following acoustic technical abbreviations and definitions are used in this manuscript:

dB	Decibels
L_Aeq(T)_	A-weighed time-average sound pressure level in dB over the time interval T (typically in hours or minutes)
L_Cpeak_	C-weighted peak sound pressure level in dB
T_mf_	Mid-frequency reverberation time in seconds, defined in Section 5.4 of AS/NZS 2107:2016 [17]
%dose	Percentage noise dose based on the 85 dB L_Aeq(8 h)_ criteria with a 3 dB exchange rate: 1 Pa^2^h = 100%
L_An_	A-weighed time-average sound pressure level in dB exceeded n% of the measurement period
