# Supplementary material for: Noise Levels and Acoustic Quality of Preschool Learning Spaces in Taiwan
Source: Int J Environ Res Public Health. 2026 Mar 23;23(3):406. doi: 10.3390/ijerph23030406 (PMC13026530; doi:10.3390/ijerph23030406)
Supplement: Supplementary file 1 [file ijerph-23-00406-s001.zip › ijerph-4028945-supplementary.pdf]

Questionnaire

Teaching spaces

一 ONE

1. What do you consider the aspects of your teaching spaces to be the most important? Please rank those given with 1 being the most important to 5 the least important

   ☐ Lighting ~
   ☐ Ventilation
   ☐ Acoustics (listening environment)
   ☐ Equipment
   ☐ Adequate space

2. How do you experience the listening environment in the rooms you teach in? Please indicate

   ☐ Comfortable
   ☐ Confusing
   ☐ Echo (reverberation)
   ☐ Harsh
   ☐ Clear
   ☐ Irritating
   ☐ Relaxing
   ☐ Other (Please describe)

2. How would you rate your room(s) teaching environment? If you teach in more than one room please indicate for each room you teach in.

Room 1	Room 2	Room 3
☐ Just right	☐ Just right	☐ Just right
☐ Good	☐ Good	☐ Good
☐ Poor	☐ Poor	☐ Poor
☐ Very poor	☐ Very poor	☐ Very poor

4. For rooms rated poor or very poor what do you consider are the most problems in the room? Please indicate

   ☐ Open plan style room
   ☐ Too much echo (reverberation
   ☐ Too much noise outside the room
   ☐ Noise produced by the children too high
   ☐ Other (please indicate)

Questionnaire

二 TWO

Noise sources generated inside the teaching rooms

1 Do you have any problems with noise created inside the rooms (including noise made by children)?

2 What amount of nose generated in the teaching spaces is made by the children?

☐ None: ☐ Some: ☐ Most : ☐ All

3 What times of the day or special events do you perceive noise to be the most excessive?

4 How much of the time during the working day (in percentage for example do you consider noise to be too high? 4/8= 50%

5 Are there any conditions (weather etc) which affect noise levels?

6 Please identify all other sources generated inside the rooms!

1 Equipment	2 Moving equipment around the room (furniture scape
3 Doors	4 Rattling windows
5 Floor Foot pounding	6 Ventilation
7 Air Con	8 Lights
9 Fans	10 Hi Fi
11 Others (indicate)	

+++++++++

三 THREE

Noise Sources Outside

1 Do you have problems with outside noise or noise from activities not associated with the centre? (Please include noise form other businesses etc if you share a building complex with other tenants or occupants.
Yes ☐ No ☐

2 Identify the sources of noise outside teaching rooms but inside Centre
☐ Noise from corridors (if applicable)
☐ Noise from other rooms or activities within centre
☐ Foot pounding/foot traffic
☐ Noise from equipment (Squeaky swings, gates, doors etc)

- ☐ Others Please explain

3 Identify the sources of noise generated outside the Centre

- ☐ Road Traffic
- ☐ Aircraft
- ☐ Rail
- ☐ Recreational vehicles and activates
- ☐ Road or earth works
- ☐ Lawn mowing
- ☐ Noise from other businesses or activities
- ☐ Dogs
- ☐ Loud music
- ☐ Other

Please explain

4 What kind of noise do you find the most intrusive and annoying?

Why?

四 FOUR

Effects of Noise on Children

1 How do you feel about the level of noise in the room?

2 How do you feel the levels of noise affects children generally?

3 Do you feel the noise is too high?

4 Do you think it may be harmful to the children?

5 Do you think it may be harmful to the teachers?

Questionnaire

五 FIVE

Effects of noise on Special needs children experiencing te following conditions.

1 Are there any students you are aware of either now or in the past few years who you have cared for who experience any one of the following disabilities? Please indicate:

- ☐ Partially sighted ☐Hearing loss ☐ Down syndrome
- ☐ Autistic Spectrum Disorder ☐ Asperger syndrome
- ☐ Pervasive developmental disorder ☐ ADHD disorder
- ☐ Giftedness (recently added)
- ☐ Other conditions resulting in delay in development, speech and communication. Please indicate

2 For each of the above disabilities you have indicated could you indicate how noise affects these children you have cared for? (i) Special need

3 Are there any sounds the child finds particularly distressing?

4 What effects of noise have you observed in these children?

5 Are there any strategies you can suggest from your practice and experience, which can be implemented to help these children?

6 In your opinion are children experiencing disability, generally more adversely affected by noise than other children?
Yes No
Comments

+++++++

六 SIX Effects of noise on teaching staff

++++

1 How do you feel noise affects teaching staff? This can be your own personal experience or that related to you by colleagues.

2 Any other comments you would like to make?

+++++

七 SEVEN Policies, procedures and further work

1 Does your centre have any formal policies and procedures in place regarding noise?

Yes ☐ No ☐

Questionnaire

If yes would you mind submitting these for consideration and dissemination as part of our study? (There are likely to be excellent individual policies, the contents of which if disseminated in a generic way could be very beneficial.)

2 What strategies do you use to minimise noise levels in your centre?

- ☐ Quiet times,
- ☐ Compulsory sleep/rest times,
- ☐ Rostering of staff between contact and non-contact duties
- ☐ Ban on loud music or activates generating excessive noise.
- ☐ Other

3 What low cost or cost effective strategies do you think could be implemented?

4 Is this an area, which needs more investigation and work?

Yes ☐ No☐

5 Is there any particular work would you like to see undertaken?

6 What other ways can we assist teaching staff and the children?

7 Please free to make any other comments or suggestions not covered in the questionnaire

幼兒園環境噪音問卷調查表

受訪者代號:
受訪日期: 西元__________年________月__________日
中心(或幼兒園)代號:
職員代號:

受訪者版本:

一、 教學空間滿意度調查

1. 請您依序排列，對您而言”最重要的教學空間”。1 代表最重要，5 代表最不重要。
   - ☐ 燈光
   - ☐ 空氣流通(通風設備，非冷氣)
   - ☐ 活動空間
   - ☐ 園區內設備
   - ☐ 音響效果 (上課時)

2. 您目前所處環境製造出的聲音讓您有什麼感覺?
   - ☐ 舒服
   - ☐ 困擾
   - ☐ 刺耳
   - ☐ 迴音
   - ☐ 煩燥
   - ☐ 清晰
   - ☐ 放鬆
   - ☐ 其他 (請描述)

3. 您對目前的教學環境有何評價? 若您分別在不同教室工作，請分別選填。

教室 1	教室 2	教室 3
☐ 非常好	☐ 非常好	☐ 非常好
☐ 良好	☐ 良好	☐ 良好
☐ 差	☐ 差	☐ 差
☐ 非常差	☐ 非常差	☐ 非常差

4. 對於給予”差”及”非常差”評價的教室，您認為最大的問題在於:
   - ☐ 開放式空間
   - ☐ 迴音太大
   - ☐ 教室外噪音過大
   - ☐ 學生太吵/製造噪音
   - ☐ 其他因素

高醫
人體試驗審查委員會
2019 -04- 19
審查通過

二、 教室內噪音來源調查

1. 您對教室內的噪音(包括學生製造的)有感到任何困擾(問題或意見)嗎?
   ☐ 有 ☐ 無
   請解釋您的選擇:

2. 在教室內有哪部份噪音是學生製造的?
   ☐ 無 ☐ 一些 ☐ 大部份 ☐ 全部

3. 您是否有注意到一天當中某些時段或特別課程中，學生會比較吵鬧?

4. 在一天工作中，您認為有多少時間（小時）是噪音最嚴重的時候? (請用百分比表示，或工作時數表示)。例如: 一天工作八小時，大約 4 小時很嚴重，4/8hr =50%

   ____

5. 是否有任何情況(例如:天氣)，影響噪音強度呢?

   ____

6. 請選擇教室內的噪音來源。

   ☐ 儀器發出的聲音
   ☐ 開關門產生的聲音
   ☐ 地板(腳步聲/高跟鞋/打球聲等)
   ☐ 通風系統產生的聲音
   ☐ 電風扇產生的聲音
   ☐ 搬動桌椅或大型儀器產生的聲音
   ☐ 敲打窗戶產生的聲音
   ☐ 空調系統產生的聲音
   ☐ 燈源產生的聲音
   ☐ 音響設備產生的聲音
   ☐ 其他 (請說明) ______________________________

三、 教室外噪音來源調查

1. 對於教室外的噪音，您是否覺得困擾?
   ☐ 有 ☐ 無

2. 請選擇哪些噪音是來自於教室外，但是仍屬於幼兒園範圍內。
   ☐ 走廊
   ☐ 其他教室或特殊活動
   ☐ 各式的走路聲音
   ☐ 儀器製造的噪音 (如: 盪鞦韆/開關門/搬動桌椅)
   ☐ 其他 (請說明) ______________________________

高醫
人體試驗審查委員會
2019 -04- 19
審查通過

3. 請選擇哪些噪音是來自於幼兒園外面。
   - ☐ 交通車輛
   - ☐ 飛機
   - ☐ 火車
   - ☐ 宣傳車或宣傳活動
   - ☐ 道路工程
   - ☐ 除草機
   - ☐ 其他商業活動
   - ☐ 狗叫聲
   - ☐ 吵鬧的音樂聲
   - ☐ 其他（請說明）________________________
4. 上述何種噪音是您覺得最吵雜的?

   ____

   為什麼?

   ____

四、 噪音對學童的影響

1. 您對於教室內的噪音程度有什麼看法?

   ____

2. 您覺得教室內的噪音對學童有什麼影響?

   ____

3. 您覺得噪音程度太高了嗎?
4. 您認為噪音對學童有傷害嗎?
5. 您認為噪音對老師有傷害嗎?

高醫
人體試驗審查委員會
2019-04-19
審查通過

五、　　噪音對殘障學童的影響

1. 您是否曾經教過特殊學生呢？請註明。

2. 對於殘障學童，噪音對他們的影響為何？請依照不同症狀分別描述。
(例: 症狀名稱 / 學生反應………)

3. 殘障學童是否會對於某些聲音感到特別痛苦或難過嗎?

4. 您覺得噪音對他們有哪些影響呢?

5. 依照您的教學經驗，是否可以請您分享您使用哪些方法及策略來協助這群特別的學童呢?

6. 您是否認為噪音對殘障學童比其他學童的影響更嚴重?

☐ 是　　☐ 否

請提出您的看法:

六、　　噪音對教職員的影響

1. 您覺得噪音對您或同事有什麼影響呢?

2. 是否有其他意見想要補充呢?

七、　　政策，規劃及未來發展

1. 請問您的工作場所是否有針對噪音制定相關政策或規劃程序（具體的工作程序指引，例如:製造必要噪音前應先請學童離開並關門）嗎?
☐ 是　　☐ 否

高醫
人體試驗審查委員會
2019-04-19
審查通過

如果有的話，是否可以請您提供相關文件，讓我們參考?

2. 請問您會使用什麼樣的方式來降低幼兒園的噪音強度呢?
   - ☐ 規定安靜時間
   - ☐ 規定學童的睡眠/休息時間
   - ☐ 編排教職員輪替處理教學與非教學職務
   - ☐ 禁止過度吵鬧的活動及音樂
   - ☐ 其他(請說明) ______________________________

3. 您認為有哪些方法可以有效的降低噪音，並且只需花費低成本。

____

4. 針對降低或控制噪音的方法，您覺得有哪方面需要更加的深入調查研究及改善呢? (請提出建議)

____

5. 您希望我們進行其他項目的研究嗎? 請說明。

____

6. 還有什麼是我們研究人員可以協助/幫助您和幼兒園學童的地方嗎?

____

7. 在問卷調查中並未提到，但您仍有其他意見或建議，請留言。

____

____

____

____

____

____

高醫 人體試驗審查委員會 2019 -04- 19 審查通過
